# Effect of UV Irradiation Time and Headgroup Interactions on the Reversible Colorimetric pH Response of Polydiacetylene Assemblies

**DOI:** 10.1021/acsomega.3c04845

**Published:** 2023-09-26

**Authors:** Gizem Beliktay, Tayyaba Shaikh, Emirhan Koca, Hande E. Cingil

**Affiliations:** † Sabanci University Nanotechnology Research and Application Center (SUNUM), Istanbul 34956, Turkiye; ‡ Sabanci University Faculty of Engineering and Natural Sciences, Istanbul 34956, Turkiye

## Abstract

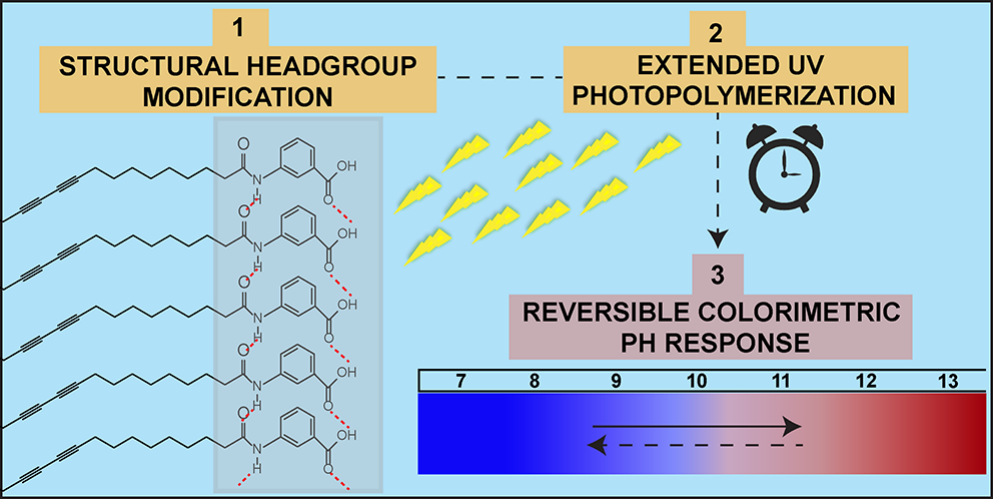

Polydiacetylenes are chromatic conjugated polymers formed upon the photopolymerization of self-assembled diacetylenes. They exhibit conformation-dependent colorimetric responses, usually irreversible, to external triggers. Here, we presented an approach to obtain a reversible colorimetric response to a pH change through structural modifications on the monomer and extended photopolymerization time. Both factors, enhanced hydrogen bond forming headgroups and longer UV exposure, impacted the rotational freedom of polydiacetylene conformation. Such a restricted conformation state reduced colorimetric response efficiency but enabled reversible colorimetric response to a pH change. These results highlight the possibility of obtaining a reversible colorimetric pH response of polydiacetylenes for customized sensing applications through monomer-level tailoring combined with tuning the photopolymerization time.

## Introduction

Supramolecular interactions are noncovalent interactions responsible for forming highly ordered, self-assembled complex molecular structures in nature.^1,2^ These interactions involve hydrogen bonds, van der Waals forces, π–π interactions, electrostatic forces, coordination bonds, and hydrophobic interactions.^3–6^ Their combined bond strength reaches comparable levels of covalent bonds when they act complementarily to form a supramolecular complex.^3–5,7^ Among them, hydrogen and ionic bonds, which bring specificity in the form of directionality and cooperativity among interacting components, are considered strong noncovalent interactions.^8^ Overall, supramolecular interactions govern the assembly/disassembly dynamics of natural and synthetic supramolecular systems such as DNA base pairs,^8^ micelles,^9^ interpolyelectrolyte complexes,^10^ hydrogels,^11^ fibers,^12^ virus-like particles,^13^ π-conjugated polymers (CPs),^14^ and many more.^15,16^ An in-depth understanding of these interactions is crucial to designing tailor-utilizing synthetic systems for specific applications. CPs are versatile tools to monitor and study supramolecular interactions.^17^ Due to their hydrophobic conjugated backbones, which commonly have aromatic structures and alkyl side chain substitutions, they engage in hydrophobic, π–π, dispersion, and hydrogen bonding interactions with the surrounding media.^3^

Due to delocalized π-electrons and conformational restrictions, several CPs exhibit chromatism. Chromatism is the optical sensitivity to conjugated backbone conformation.^3^ Any change in pH, temperature, solvent, and ionic strength of the surroundings or any interaction with analytes alters the backbone conformation of chromatic CPs.^18^ Monitoring these changes in the optical response of chromatic CPs offers valuable insights into the fundamental mechanisms of supramolecular interactions.^19–23^ Polydiacetylenes (PDAs), a class of chromatic CPs, are obtained by γ or UV photopolymerization of self-assembled amphiphilic diacetylene (DA) monomers in aqueous conditions.^24^ For a successful cross-linking, the 1,4-addition of alternating *ene-yne*, self-assembled DAs should align with a tilt angle of 45° and a spatial spacing of 5 Å among adjacent monomers.^24,25^ This is provided by van der Waals dispersion interactions between the hydrophobic alkyl side chains.^26^

Solutions containing PDA turn blue once photopolymerized due to the π–π* transition in the alternating bonds along the conjugated backbone.^25^ The blue solution of PDAs transitions to red if DA monomers experience external stress caused by changes in inter- and intramolecular interactions. To release the stress, DAs undergo a realignment of their conjugated backbone conformation from planar to twisted nonplanar.^27^ This transition is irreversible because the red phase is a lower energy, thermodynamically stable state than the metastable blue phase.^28,29^ PDAs in planar conformation are nonfluorescent, whereas their twisted nonplanar red-phase conformation makes the system fluorescent. Therefore, PDAs have two optical response channels: a visual colorimetric blue-to-red change and a fluorescence off-to-on change. The supramolecular interactions that PDAs engage with directly affect these optical responses. Any factor, such as the structure of DA monomers in terms of alkyl chain and spacer length, headgroup functionalities, and photopolymerization processes, including the method and exposure durations, modifies inter- and intramolecular interactions.^26,30–34^ This provides a platform to utilize PDAs as colorimetric sensors for changes in the surrounding vicinity, while supramolecular interactions are monitored in a self-assembling system. Recently, we showed how the initial conformation of PDA vesicles, especially when it is a phase other than blue, influences their colorimetric response to various triggers in solution.^35^

The pH of an environment is a crucial factor for processes in biological and chemical reactions.^36^ Depending on the headgroup, deprotonation or protonation promotes the ionic repulsive interactions in PDAs, resulting in colorimetric pH responsiveness.^30,31^ Their irreversible colorimetric response has been exploited to construct PDA-based sensors, especially for food sensing applications.^37,38^ A few examples of reversible colorimetric responses to changes in temperature^26,39^ and pH^28,30,40–42^ have been reported. Monocarboxylic headgroup bearing PDAs lack required strong headgroup interactions, i.e., H-bonds, to facilitate the molecular reorganization of polymer chains back to their original planar conformation upon removing external triggers.^18^ Including functional groups that enhance H-bond interactions among headgroups was a strategy to bring reversible thermochromism to PDA vesicles in nonaqueous solvents,^43^ films,^30^ and vesicles^44^ or with other constituents, such as fluorescent dyes.^40^

We present here a unique approach to obtaining a reversible colorimetric pH response of PDA assemblies in aqueous conditions by modifying headgroups and tuning the photopolymerization duration of DA monomers. We worked on two DA monomers with different lengths of alkyl chains and the same headgroup functionalities. We modified them by substituting their carboxylic acid headgroup with carboxy-*meta*-anilido benzoic acid (BzA) functionalities. Such modification enhances the hydrogen bonding capabilities among headgroups within the self-assembled structure. Enhanced hydrogen bonding is required as an additional restorative force to reverse back to the thermodynamically unfavorable blue phase.^42^ We then photopolymerized both modified and unmodified DAs at durations varying from 1 to 20 min and monitored their pH response within pH 7–13 with absorption, fluorescence, and light scattering methods.

Results indicated that structural modification on the monomer and long photopolymerization time impacted PDAs’ conformational freedom within the assembly. This limitation revealed itself when they were exposed to pH change. Even though they responded to pH change colorimetrically, we calculated a reduced, ∼ 75%, colorimetric percentage (response efficiency) compared to PDAs produced in a shorter photopolymerization time. Such reduced response efficiency indicates a limited conformation transition from the planar blue phase to the twisted nonplanar red phase, and it was instrumental in achieving a reversible colorimetric response. We believe that upon pH change, the backbone conformation of these PDAs does not completely adopt a twisted nonplanar red phase conformation but a mixture of nonplanar (major) and planar (minor) conformations. Such a mixture of backbone conformations provides a certain amount of freedom for chains to either return to the planar blue conformation or transition to the intermediate purple conformation. These results shed new light on the combined effects of UV photopolymerization time and enhanced headgroup interactions on the color transition pH of PDA assemblies. Utilizing these parameters can open possibilities for tuning the color transition pH of PDA systems with reversibility options for specific applications.

## Experimental Section

### Chemicals

10,12-Pentacosadiynoic acid (PCDA), 10,12-tricosadiynoic acid (TCDA), oxalyl chloride, anhydrous tetrahydrofuran (THF), dimethyl sulfoxide, and PTFE filters (0.45 μm) were purchased from Sigma-Aldrich. Triethylamine, acetone, and potassium hydroxide were purchased from Merck. Dimethylformamide (DMF), anhydrous dichloromethane, and methanol were purchased from Scharlab. 3-Aminobenzoic acid was purchased from AlfaAesar, dichloromethane was purchased from ACS Reagent, hydrochloric acid was purchased from Carlo Erba Reagents, and ethanol was purchased from Isolab Chemicals. Poly(vinylidene difluoride) (PVDF) filters (0.45 μm) were purchased from Whatman. All chemicals were used as received, unless otherwise stated. Milli-Q water (18.2 MΩ cm) was used to prepare polymer solutions.

### Characterization Methods

^1^H and ^13^C nuclear magnetic resonance (NMR) spectra were recorded on a Bruker AVANCE NEO 700 NMR Spectrophotometer. Fourier transform infrared (FTIR) spectra were obtained with a Shimadzu IRAffinity-1S equipped with an attenuated total reflectance (ATR) sampling accessory from Pike Technologies. The ATR–FTIR spectra were recorded over the 4000–1000 cm^–1^ range with a resolution of 4 cm^–1^ and 20 total scans. Thermal properties of monomers were detected using differential scanning calorimetry (DSC) from a TA Instruments DSC Q2000 and a Mettler Toledo DSC3 under nitrogen gas at 5 °C/min heating and cooling rate. Thermogravimetric analysis (TGA) and derivative thermogravimetry (DTG) were done using a Netzsch STA TG/DTA 449C Jupiter instrument under nitrogen gas, ramping from 25 to 900 °C, with a heating rate of 10 °C/min. UV–vis absorption spectra were collected by using a PG Instruments T80+ spectrophotometer. Steady-state fluorescence emission measurements were performed on a Shimadzu RF-6000 fluorescence spectrophotometer. Dynamic light scattering (DLS) experiments were performed by using a Malvern Zetasizer NANO ZS at 173° with a 633 nm laser. For scanning electron microscopy (SEM) imaging, 4 μL samples were drop-cast onto silicon wafer pieces and dried at RT overnight. Then, the samples were sputter-coated (Cressington 108) with Au/Pd for 120 s with a 40 mA current. SEM micrographs were taken using the mixed signal of secondary and in-lens electron detectors with a mixing ratio of 0.60 of field emission SEM (Zeiss Supra 35VP FE-SEM). The samples were imaged by using a 3 kV accelerating voltage. All measurements were performed at 21 °C unless otherwise stated.

### Synthetic Procedures

#### Monomer Modifications: PCDA-*m*BzA

We slightly altered an earlier protocol to obtain the carboxy-substituted anilido DA, 3-(pentacosa-10,12-diynamido) BzA (PCDA-*m*BzA) monomer.^30^ PCDA (100.5 mg, 0.27 mmol) was dissolved in 4 mL of anhydrous DCM in a round-bottom flask wrapped up with aluminum foil to ensure darkness in the reaction vessel. The solution was flushed with nitrogen before the dropwise addition of oxalyl chloride (300 μL) and anhydrous DMF. The reaction mixture was refluxed under nitrogen and stirred overnight at 45 °C. DCM was evaporated via a rotary evaporator (Hei-VAP Core) to concentrate the solution. The product (PCDA-Cl) was dissolved in 1 mL of anhydrous DCM and combined with 3-amino BzA (45.1 mg, 0.33 mmol) separately in 3 mL of anhydrous THF. Subsequently, 300 μL of triethylamine was added to the reaction mixture and stirred at room temperature under nitrogen for 24 h. The solvents were evaporated via a rotary evaporator before fully redissolving the product (PCDA-*m*BzA) in 2.5 mL of THF. To purify PCDA-*m*BzA, a solution mixture containing methanol, acetone, 0.1 M HCl, and Milli-Q (1:1:1:1) volume ratio was prepared. The product was dispersed in 40 mL of this mixture and then centrifuged. After this process was repeated three times, the precipitate was collected and dried in a vacuum oven at 45 °C. The monomer was stored in a glass vial wrapped in aluminum foil at 4 °C. (PCDA-*m*BzA, 48.6 mg). ^13^C NMR (176 MHz, DMSO-*d*_6_, 295 K): δ = 174.51, 171.92, 171.48, 167.21, 139.50, 131.22, 128.92, 123.75, 123.03, 119.71, 78.01, 77.99, 65.36, 36.71, 36.41, 34.76, 33.66, 32.34, 31.33, 29.03, 28.97, 28.88, 28.74, 28.69, 28.63, 28.53, 28.40, 28.36, 28.34, 28.22, 28.20, 28.18, 27.73, 27.70, 25.04, 24.67, 24.48, 22.13, 18.29, 18.28, 13.99. ^1^H NMR (700 MHz, DMSO-*d*_6_, 295 K): δ = 11.99, 10.04, 8.22, 7.81, 7.59, 7.40, 2.27, 2.18, 1.60, 1.59, 1.57, 1.49, 1.48, 1.47, 1.44, 1.43, 1.42, 1.31, 1.27, 1.25, 1.24, 1.24, 1.23, 0.85.

#### TCDA-*m*BzA

The same procedure was followed to obtain 3-(tricosa-10,12-diynamido) BzA (TCDA-*m*BzA, 56.3 mg). ^13^C NMR (176 MHz, DMSO-*d*_6_, 295 K): δ = 174.51, 171.92, 171.53, 171.44, 167.20, 139.55, 139.45, 131.19, 128.92, 123.75, 123.09, 122.99, 119.75, 119.66, 78.01, 65.36, 36.71, 36.41, 36.36, 34.76, 33.65, 32.34, 31.32, 29.03, 28.94, 28.90, 28.80, 28.79, 28.73, 28.69, 28.62, 28.52, 28.41, 28.36, 28.22, 28.19, 27.73, 27.71, 25.04, 24.67, 24.48, 22.12, 18.28, 13.99. ^1^H NMR (700 MHz, DMSO-*d*_6_, 295 K): δ = 12.92, 10.04, 8.22, 7.81, 7.59, 7.40, 2.30, 2.27, 2.18, 1.60, 1.58, 1.57, 1.45, 1.44, 1.43, 1.42, 1.32, 1.31, 1.29, 1.28, 1.26, 1.23, 0.85.

#### Preparation of PDA Assemblies in Solution

To prepare PDA assemblies in solution, we followed a method described earlier.^35^ PCDA (2.3 mg, 6.1 μmol) was dissolved in 1 mL of THF. Solutions were filtered through a 0.45 μm PVDF syringe filter (Whatman), and the solvent was evaporated under a direct flow of nitrogen to form a thin film at the interior of the glass vial. 6.0 mL of Milli-Q water at 80 °C was added to obtain a 1 mM solution. After vortexing for 2 min, the mixture was transferred to a sonic bath (Elmasonic S30 H) at 80 °C and dissolved thoroughly by vortex-sonication cycles for 1 h. The solution vial was wrapped in aluminum foil, cooled to room temperature, and stored at 4 °C overnight. Photopolymerization was performed under UV irradiation at 254 nm in a UV cabinet (8W, CAMAG UV Cabinet 4) after the monomer solutions were acclimated to room temperature. The same procedure was followed to obtain PDA assemblies from monomers, TCDA, PCDA-*m*BzA, and TCDA-*m*BzA with the same molar ratio. For the TCDA monomer, ethanol was used; for the PCDA-*m*BzA and TCDA-*m*BzA monomers, DMSO was used as the solvent. PVDF syringe filters (0.45 μm) were used for TCDA, and PTFE syringe filters (0.45 μm, ISOLAB) were used for the PCDA-*m*BzA and TCDA-*m*BzA solutions to remove undissolved monomers before photopolymerization.

### Experimental Procedures

#### Photopolymerization Experiments

Monomer solutions of PCDA, TCDA, PCDA-*m*BzA, and TCDA-*m*BzA were exposed to UV irradiation at 254 nm for 1, 3, 5, 10, 15, and 20 min. The photos of polymer solutions were taken directly without any dilution. For the UV–vis spectroscopy measurements, the polymer solutions were diluted to obtain the highest absorbance intensity from the sample prepared under 20 min UV exposure in the range of 0.8–1.0. That dilution factor is used for all samples of the same PDA but varies among different PDAs.

#### pH Response Experiments

The pH response experiments were conducted with 600 μL solutions of poly(PCDA), poly(TCDA), poly(PCDA-*m*BzA), and poly(TCDA-*m*BzA). The pH of the solutions was monitored using a pH meter (pH electrode InLab Micro, Mettler-Toledo). KOH solutions (0.1–5 M) were added dropwise to increase the pH while maintaining the total added volume between 0.2 and 5 μL to prevent dilution effects. After each KOH addition, the solution was vortexed for 3 s, and the pH was measured. Additional KOH solution was added if necessary, and the vortex and pH measurement cycles were repeated until the target pH was reached. Subsequently, the solution was allowed to equilibrate for 5 min before conducting UV–vis, fluorescence, and DLS measurements. We avoided the addition of HCl to adjust the pH during pH response experiments to prevent an increase in the ionic strength.

For the pH reversibility tests, initially, we raised the solution pH from 7 to 13 by adding 5 M KOH dropwise. To drop the solution pH back to 7, we added HCl (0.01–0.5 M) dropwise into the solution while keeping the total added volume below 5 μL, vortexed gently for 3 s, and then measured the pH after each addition. After the pH reached 7, we recorded the absorption spectra immediately.

The colorimetric response was calculated by the following formula:^35^ CR (%) = [(PB_initial_ – PB_final_)/PB_initial_] × 100. PB_initial_ is the before exposure values, and PB_final_ is the after exposure values for the system’s percent blue (PB) calculated from PB = Abs_blue_/(Abs_blue_ + Abs_red_). Abs_blue_ represents the blue-phase-attributed absorbance peak maximum intensity around 640 nm, and Abs_red_ represents the red-phase-attributed absorbance peak maximum intensity around 540 nm.

## Results and Discussion

### Monomer Modifications

**Figure 1:**
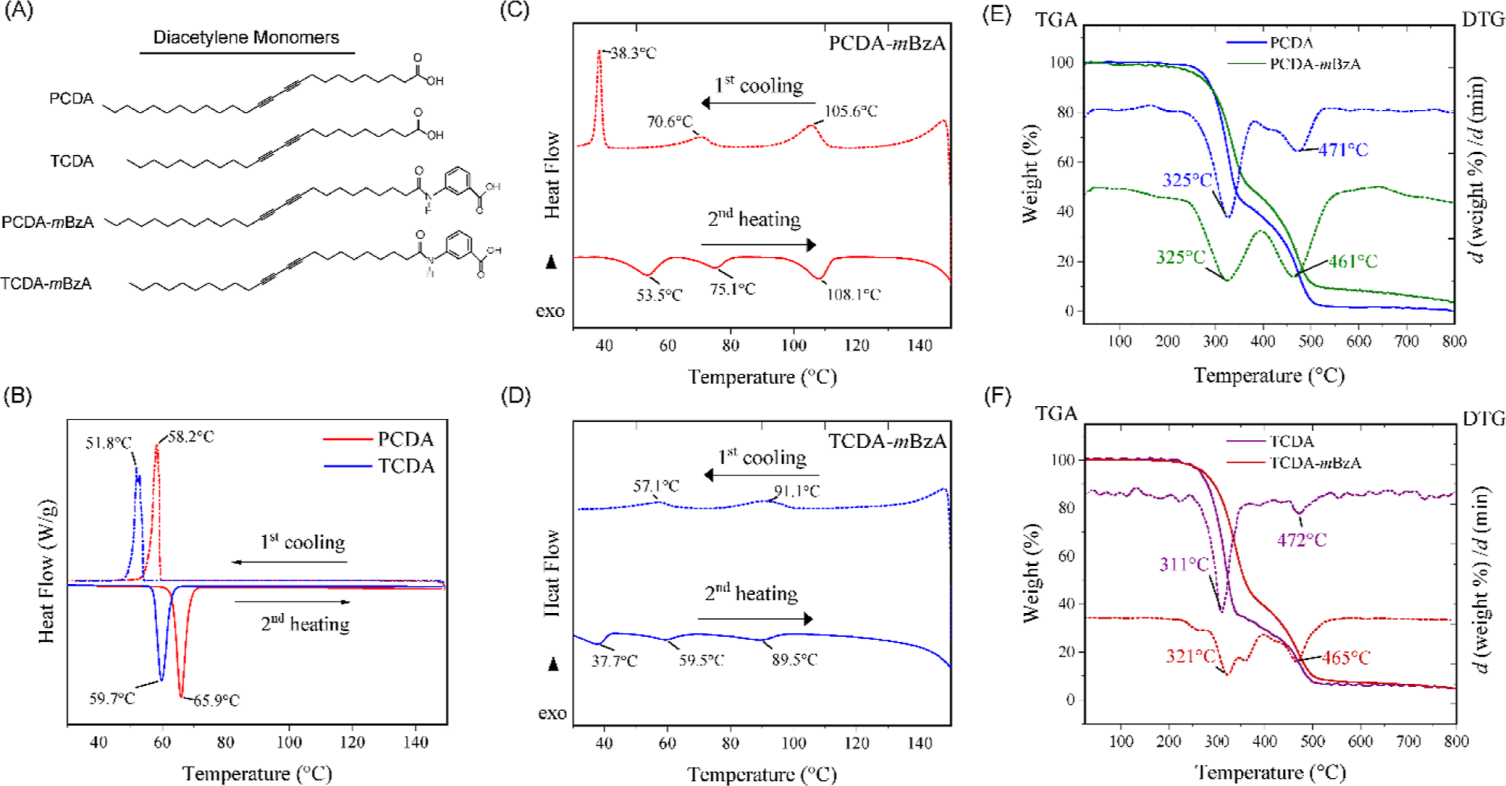
(A) Chemical structures of DA monomers used in this study. DSC thermograms of second heating and first cooling of (B) PCDA and TCDA, (C) PCDA-*m*BzA, and (D) TCDA-*m*BzA. TGA curves (left) and DTG peaks (right) of (E) PCDA and PCDA-*m*BzA and (F) TCDA and TCDA-*m*BzA.

We modified commercially available PCDA and TCDA by coupling carboxy-substituted *meta*-anilide with DA headgroups to obtain PCDA-*m*BzA and TCDA-*m*BzA monomers (Figure 1A). DA monomers were first treated with a chlorinated agent to yield DA*-Cl* and then reacted with 3-aminobenzoic acid. The detailed synthesis procedures are given in the Experimental Section, and NMR and FTIR spectra are available in the Supporting Information (Figures S1–S5). NMR spectroscopy results confirmed the substitution of *m*BzA moieties on DA monomers with new peaks between 12.0 and 7.0 ppm in ^1^H NMR and 173.0–115.0 ppm in ^13^C NMR. FTIR spectra of the *m*BzA-substituted monomers show the emergence of new vibrational bands at ∼3267, 1657, 1593, and 1543 cm^–1^ which correspond to aromatic secondary NH stretching, C=O stretching at the amide group, C=C stretching at the phenyl group, and CNH bending, respectively.^26^ Furthermore, we assessed the common DA vibrational bands such as the asymmetric (*v*_a_) and symmetric (*v*_s_) stretching vibrations of the alkyl side chains (CH_2_) that are present at around 2918–2847 cm^–1^, respectively. The carbonyl (C=O) stretching at the terminal carboxylic group appears at ∼1692 cm^–1^. With all of these vibrational bands, we confirmed the substitution of *m*BzA moieties on DA monomers.

### Thermal Properties of Modified Monomers

We investigated the thermal properties of modified DA monomers compared to the unmodified ones to assess the effect of bulky headgroup substitution on the strength of intermolecular interactions by DSC and TGA. Figure 1B shows the DSC thermograms of monocarboxylic PCDA and TCDA monomers obtained from the second heating cycle and the first cooling cycle. The melting temperatures (*T*_m_) recorded for PCDA and TCDA monomers are 65.9 and 59.8 °C, respectively, in line with earlier reports.^31,45^ The enthalpy change (Δ*H*_endo_) of the TCDA monomer is calculated as 60.4 kJ/mol, which is lower than the Δ*H*_endo_ of PCDA, 71.4 kJ/mol. Since both monomers possess the same monocarboxylic headgroups, lower *T*_m_ and Δ*H*_endo_ indicate that the shorter alkyl tail of TCDA leads to weaker dispersion forces in TCDA assemblies. Thus, the thermal energy barrier to dissociate the TCDA system is lower than that of the longer alkyl tail-possessed PCDA. Figure 1C,D shows the DSC thermograms of headgroup-modified monomers obtained from the second heating and the first cooling cycle. We see that the change of headgroup changes the thermal properties of DA monomers dramatically. Modified monomers exhibit multiple transition temperatures with broad peaks, indicative of the enantiotropic crystalline phases in both systems.^31,38,46^Table 1 summarizes the transition temperatures and the corresponding enthalpy changes.

**Table 1:** Thermal Properties of DA Monomers Upon First Cooling and Second Heating Cycles^a^

	second heating		first cooling	
DA monomers	*T*_m_ (°C)	Δ*H*_endo_ (kJ/mol)	*T*_c_ (°C)	Δ*H*_exo_ (kJ/mol)
PCDA	65.9	72.1	58.2	72.4
TCDA	59.7	60.5	51.8	60.3
PCDA-*m*BzA	53.5, 75.1 (T_LC_)	3.8	38.3, 70.6 (T_LC_)	5.0
	108.1 (*T*_m_)	3.3	105.6 (*T*_c_)	3.3
TCDA-*m*BzA	37.7, 59.5 (T_LC_)	1.2	57.1 (T_LC_)	0.8
	89.5 (*T*_m_)	1.4	91.1 (*T*_c_)	1.4

a *T*_m_: melting temperature, Δ*H*_endo_: endothermic enthalpy change, *T*_c_: recrystallization temperature, Δ*H*_exo_: endothermic enthalpy change, T_LC_: liquid-crystal transition temperature.

The transition peaks in the second heating of PCDA-*m*BzA appeared at 51.4, 72.2, and 106.9 °C, with corresponding Δ*H*_endo_ values of 2.6, 1.2, and 3.3 kJ/mol, respectively (Figure 1C). Similarly, TCDA-*m*BzA exhibited broad peaks at 38.8, 59, and 92.4 °C, along with the corresponding Δ*H*_endo_ values of 0.6, 0.6, and 1.4 kJ/mol (Figure 1D). Transitions at low temperatures may result from side chain relaxation, while those at higher temperatures can be attributed to rigid core movement.^47,48^ We believe that the low enthalpy values in modified monomers are due to the clearing enthalpies of -*m*BzA moieties.^46^ Therefore, the H-bond formation among headgroups plays a key role in the liquid-crystal phase formation of DA monomers where the strength of these H-bonds varies in a wide range, leading to broader transition endotherms in DSC curves.^38,49^ The polymorphic structure of modified DA monomers is further confirmed by the first cooling curves. In PCDA-*m*BzA, the first cooling exhibits three exothermic transitions, indicating recrystallization near the temperatures of crystalline site melting (Figure 1C).

For TCDA-*m*BzA, the first cooling exhibits two broad exothermic transitions, suggesting incomplete recrystallization (Figure 1D).^46^ Since both modified monomers have the same -*m*BzA headgroup, the difference could be attributed to the combined effect of the alkyl tail and the headgroup modification. We performed TGA to follow the decomposition patterns and compare the thermal stabilities of modified and unmodified DA monomers. Figure 1E,F shows the TGA curves on the left and the DTG analysis peaks on the right axis for PCDA with PCDA-*m*BzA and TCDA with TCDA-*m*BzA, respectively. Results show two decomposition steps for all DAs, in line with earlier reports.^50^ The first major mass loss, 58%, occurred within the range of 240–380 °C, with the maximum at 327 °C, and the second one, 38.5%, between 380 and 520 °C, with the maximum at 471 °C for PCDA. For PCDA-*m*BzA, we recorded a mass loss of 50.7% within the 230–390 °C range, with the maximum at the same temperature as PCDA. The second decomposition step had 37.7%, which appears to be within the range of 390–550 °C, with a maximum at 461 °C, which is 10 °C lower than that for unmodified PCDA. In the first decomposition step of TCDA, 63.2% mass loss occurred within the 240–350 °C range, maximum at 311 °C, whereas 57.9% mass loss was recorded for TCDA-*m*BzA within the 230–390 °C range, maximum at 321 °C, which is 10 °C higher than that for unmodified TCDA. The second decomposition step had 28.3% mass loss within 350–520 °C, with a maximum of 472 °C for TCDA, whereas for TCDA-*m*BzA, it is 390–550 °C with a maximum at 463 °C, which is 10 °C lower than that for TCDA, with 32.3% mass loss. Results with lower mass loss for almost the same temperature ranges for headgroup-modified DAs indicate that the *m*BzA moiety indeed improved their thermal stability. These results further support the DSC results, indicating enhanced intermolecular interactions with additional H-bonds among bulky *m*BzA headgroups in modified DAs.

### Photopolymerization Efficiency of DA Monomers

**Figure 2:**
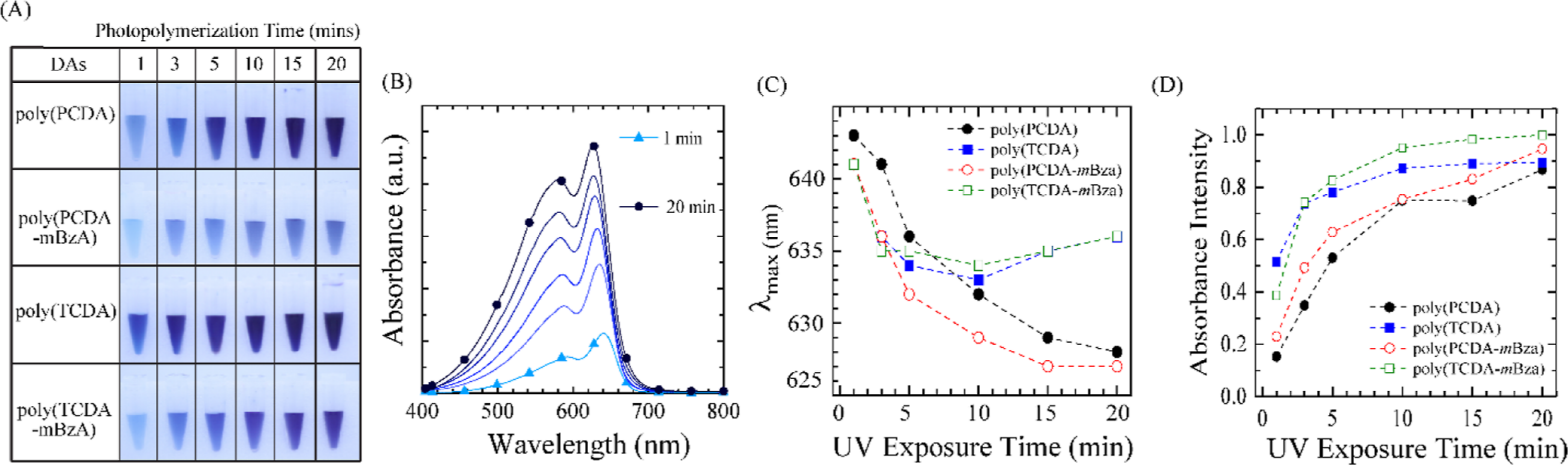
(A) Photos of PDA solutions obtained after varying photopolymerization durations from 1 to 20 min. (B) Corresponding UV–vis absorption spectra of poly(PCDA-*m*BzA). The change in (C) absorption maximum (λ_max_) and (D) absorbance intensity at corresponding λ_max_ of all PDA solutions prepared at different UV exposure (photopolymerization) times.

The colorless aqueous solutions of PCDA, TCDA, PCDA-*m*BzA, and TCDA-*m*BzA monomers were photopolymerized under UV exposure for various durations. Figure 2A shows the pictures of PDA solutions obtained after 1, 3, 5, 10, 15, and 20 min of photopolymerization. Blue color solutions confirmed the generation of *ene-yne*-conjugated chains via successful polymerization.^25^ The alterations or substitutions of other moieties to the DA monomers alter the molecular packing of assemblies, thus affecting the photopolymerization efficacy.^51,52^ The darker blue solutions indicate a higher monomer-to-polymer conversion.^51,53^ PDAs exhibit distinct differences in color brightness at already 1 min of UV exposure. The poly(TCDA) solution appeared noticeably darker than the rest, indicating a higher photopolymerization efficiency. All PDA solutions exhibited darkening of the blue color upon longer UV exposures. We recorded the UV–vis absorption spectra of all solutions prepared at different photopolymerization durations. The absorption spectra of poly(PCDA-*m*BzA), exposed to UV irradiation from 1 min up to 20 min, are given in Figure 2B, whereas the spectra of other PDA solutions are given in Supporting Information Figure S6. The absorption spectra exhibited the typical blue-phase PDA absorbance characteristics^31^ with a maximum absorbance at ∼640 nm and a peak around ∼590 nm. Longer exposures to UV irradiation caused an increase in the absorption intensity while broadening the spectra, similar to earlier reports.^27,54^ There is also a blue shift of the absorption maximum (λ_max_) at prolonged UV exposures.

Figure 2C shows the change in λ_max_ observed at different photopolymerization durations for all PDA solutions. The blue shift occurs gradually from ∼641 to 627 nm when the photopolymerization duration is extended from 1 to 20 min for poly(PCDA) and poly(PCDA-*m*BzA). However, poly(TCDA) and poly(TCDA-*m*BzA) systems do not follow the same gradual blue shifting trend; the blue shift to 634 nm occurs after 3 min of photopolymerization and changes only ±1 nm at longer photopolymerization durations.

To assess whether this trend correlates with the amount of monomer-to-polymer conversion in solution, we plotted the actual absorbance intensities (*I*_abs_) recorded for PDAs at each photopolymerization time in Figure 2D. We see that *I*_abs_ which is directly related to the concentration of absorbing species, PDAs, in solution increases in all PDA solutions upon UV irradiation. At 1 min UV exposure, the *I*_abs_ value of poly(TCDA) solution is highest, followed by that of poly(TCDA-*m*BzA). Most of their *I*_abs_ increase occurred within 10 min of UV irradiation. After that, we observed a plateau in the absorption intensity, which might indicate that the threshold for conversion of all monomers into polymers has been reached. The *I*_abs_ values of poly(PCDA) and poly(PCDA-*m*BzA) solutions are almost the same after 1 min UV exposure and significantly lower than those of the short alkyl chain possessing counterparts. Their intensity increased gradually, almost 5-fold, with no plateau observed from 1 to 20 min UV irradiation. Therefore, we believe that 20 min UV exposure is not yet the full conversion threshold for long alkyl chain PDA systems.

The absorption intensity of poly(TCDA) and poly(TCDA-*m*BzA) solutions correlates with the λ_max_ value obtained for the same system at prolonged UV irradiation in Figure 2C. Similarly, the gradual blue shift of λ_max_ in poly(PCDA) and poly(PCDA-*m*BzA) solutions also correlates with the gradually increasing *I*_abs_ due to continuing polymerization. These results indicate that monomer-to-polymer conversion under UV irradiation occurs more quickly in shorter alkyl side chains possessing PDAs. The reason might be the weaker van der Waals interactions among adjacent DA monomers, which cause higher packing density^27,55^ and ease photopolymerization. Moreover, the monocarboxylic monomers lack the bulky aromatic headgroup that *m*BzA-modified DAs have. Such a bulky structure increases the intermolecular distance between DA monomers and slows down the photopolymerization.^32^

### Morphology and Size Distribution of PDA Assemblies

**Figure 3:**
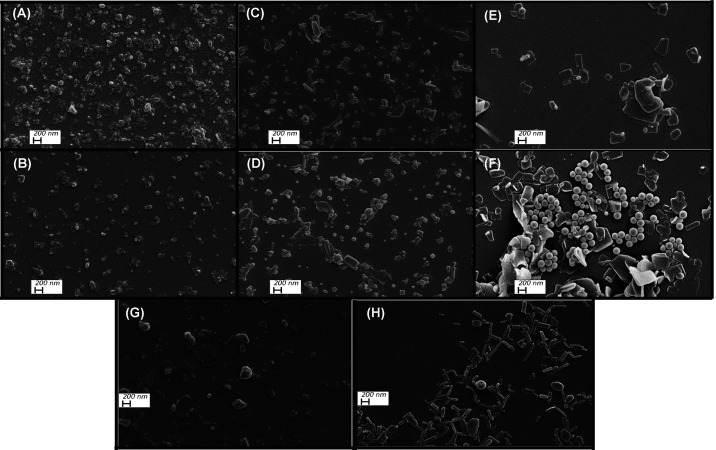
SEM images of poly(PCDA) photopolymerized at (A) 3 min and (B) 20 min; poly(PCDA-*m*BzA) photopolymerized at (C) 3 min and (D) 20 min; poly(TCDA) photopolymerized at (E) 3 min and (F) 20 min; and poly(TCDA-*m*BzA) photopolymerized at (G) 3 min and (H) 20 min.

We imaged the microstructures of PDAs prepared under 3 and 20 min of UV photopolymerization with SEM (Figure 3). Since SEM images contain dried PDAs on silicon wafers, the interpretation was done carefully considering the drying effects, such as aggregation and distorted square shapes, reported earlier.^31^Figure 3A shows that poly(PCDA) assemblies prepared under 3 min UV exposure are largely in spherical shape (*d* ∼ 85 nm), which does not change significantly when poly(PCDA) was exposed to prolonged UV exposure for 20 min, as shown in Figure 3B. There are rod-like shapes apparent in the system but limited in number. Assemblies formed by poly(PCDA-*m*BzA) under short UV exposure in Figure 3C show spherical shape similar to poly(PCDA); however, the ones exposed to 20 min UV exposure in Figure 3D show a few plate-like assemblies alongside the predominant spherical shape (*d* ∼ 100 nm). The SEM images of shorter alkyl tails possessing poly(TCDA) and poly(TCDA-*m*BzA) exhibit significantly different morphologies and spherical, rod-like, and sheet-like structures at both photopolymerization durations. Poly(TCDA) produced in 3 min photopolymerization predominantly shows irregular square shape with plates as shown in Figure 3E, whereas the ones exposed to longer UV exposure in Figure 3F exhibit the most heterogeneous system in terms of structure. It exhibits large, uniform spherical objects (*d* ∼ 150 nm) alongside large plate-like structures. The size of the rods and plates gets bigger and dominates the system. The obtained product is poly(TCDA-*m*BzA). Figure 3G shows poly(TCDA-*m*BzA) obtained at 3 min with large rods and plates as dominant structures. At longer photopolymerization, we see much smaller, uniform, rod-like structures in Figure 3H. The irregular, nonspherical shapes of PDAs, especially the modified ones, were reported earlier.^56,57^ We prepared all of our DA solutions by following the hydration preparation method and followed the same photopolymerization technique to obtain polymerized assemblies. The final morphology of those assemblies also depends on the preparation method,^58,59^ and one method might not fit for all DAs to obtain one uniform structure. That might explain the significant differences between short- and long-alkyl-tail-possessed PDAs, where the strength of dispersion interactions is essential for the assembly formation changes. Overall, SEM results suggest that the photopolymerization duration, headgroup alterations, and side chain length altogether determine the structure of assemblies formed by amphiphilic DA monomers.

Although SEM is a powerful tool to study the microstructure of systems, its images represent dried and collapsed structures of otherwise highly hydrated systems.^27^ DLS is a complementary tool for studying such systems in a nondestructive way. However, DLS also poses limitations to studying our system due to its assumption of spherical particle shapes to derive size and distribution information. Nonetheless, we performed DLS experiments on PDAs and checked the changes in the size distribution at various UV exposures. Figure S7 shows a single-size distribution with a mean hydrodynamic radius (*R*_h_) = 68 nm and polydispersity index (PDI) = 0.27 for poly(PCDA-*m*BzA) photopolymerized in 1 min. The mean *R*_h_ gradually reduced to 60 nm while retaining its single-size distribution but becoming more polydisperse, PDI = 0.38, when the photopolymerization duration was increased to 20 min. The size distribution results of other polymer assemblies are given in Supporting Information Table S1. PDAs photopolymerized in 1 min exhibited a single-size distribution with varying *R*_h_, 74 nm for poly(TCDA), 152 nm for poly(TCDA-*m*BzA), and 46 nm for poly(PCDA). The tail length variations within the hydrophobic region significantly influence the packing parameter.^60^ Specifically, PDAs with the identical headgroup but shorter alkyl tails exhibit a higher packing parameter, leading to larger vesicular structures consistent with the previous reports.^45^ The larger polymer assemblies obtained from *m*BzA-substituted monomers are due to the bulky BzA headgroups. The bulky headgroups expand the distance between DA molecules during the molecular packing, leading to larger size formations.^32^ At prolonged photopolymerization, PDI of PDAs obtained from TCDA and TCDA-*m*BzA monomers did not show a significant difference, while *R*_h_ reduced from 74 to 64 nm and from 152 to 125 nm, respectively. However, PDI has increased dramatically for poly(PCDA), similar to its modified version.

### Colorimetric pH Response of PDA Assemblies

**Figure 4:**
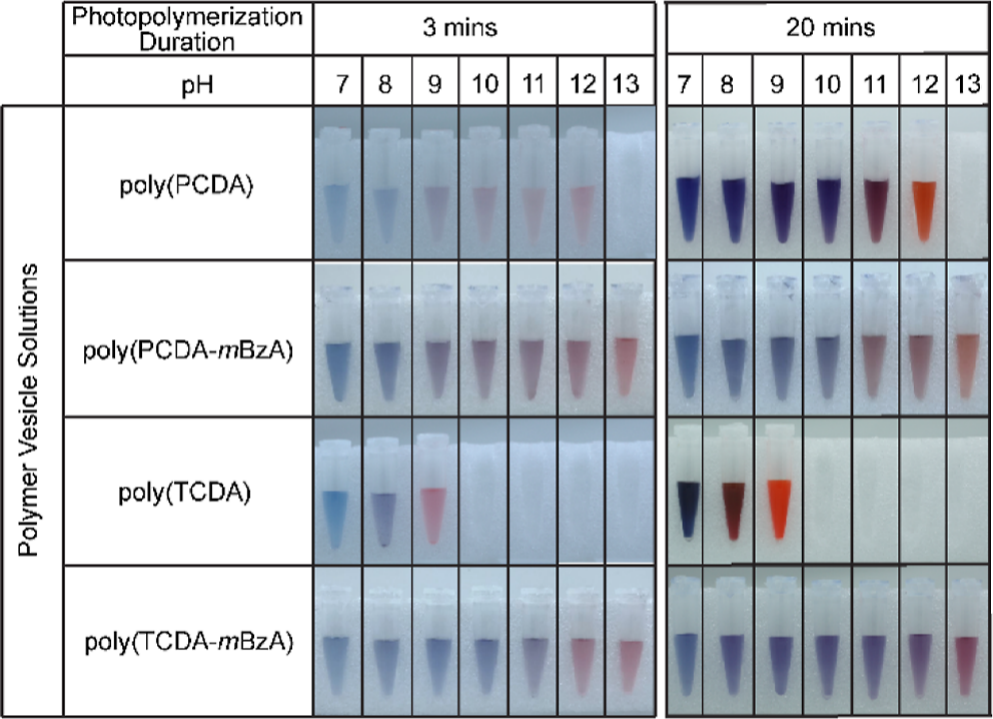
Photos show the colorimetric response of PDA solutions photopolymerized in 3 and 20 min to pH change from 7 to 13.

To study how the photopolymerization duration of PDA assemblies affects their pH response, we prepared PDAs obtained after 3 and 20 min of photopolymerization durations. Since *m*BzA-substituted and unsubstituted monomers used in this study contain carboxyl functional headgroups, colorimetric response to pH occurs at basic conditions.^44^ The deprotonation of carboxylic acid headgroups under basic conditions creates repulsive forces that break the hydrogen bonds among the headgroups and exert stress on the polymer backbone. Above a certain threshold pH, the repulsive forces overcome the dispersion forces among the alkyl side chains of PDA and force for a structural realignment to reduce strain.^44^ Thus, the conformational transition occurs from the planar, blue phase to the twisted, nonplanar, red-phase conformation.^31^ After preparing polymer solutions, we increased their pH by adding KOH. We recorded the images and UV–vis absorption spectra of PDA solutions to follow the colorimetric response to the pH change. Figure 4 shows the images of PDAs obtained after 3 and 20 min of photopolymerization while pH varies from 7 to as high as 13. All PDA solutions exhibited colorimetric responses to pH as a change from blue to purple and finally to red. The visual images show clear differences in the pH values that PDAs transition from planar, blue phase to nonplanar, red-phase conformation.

**Figure 5:**
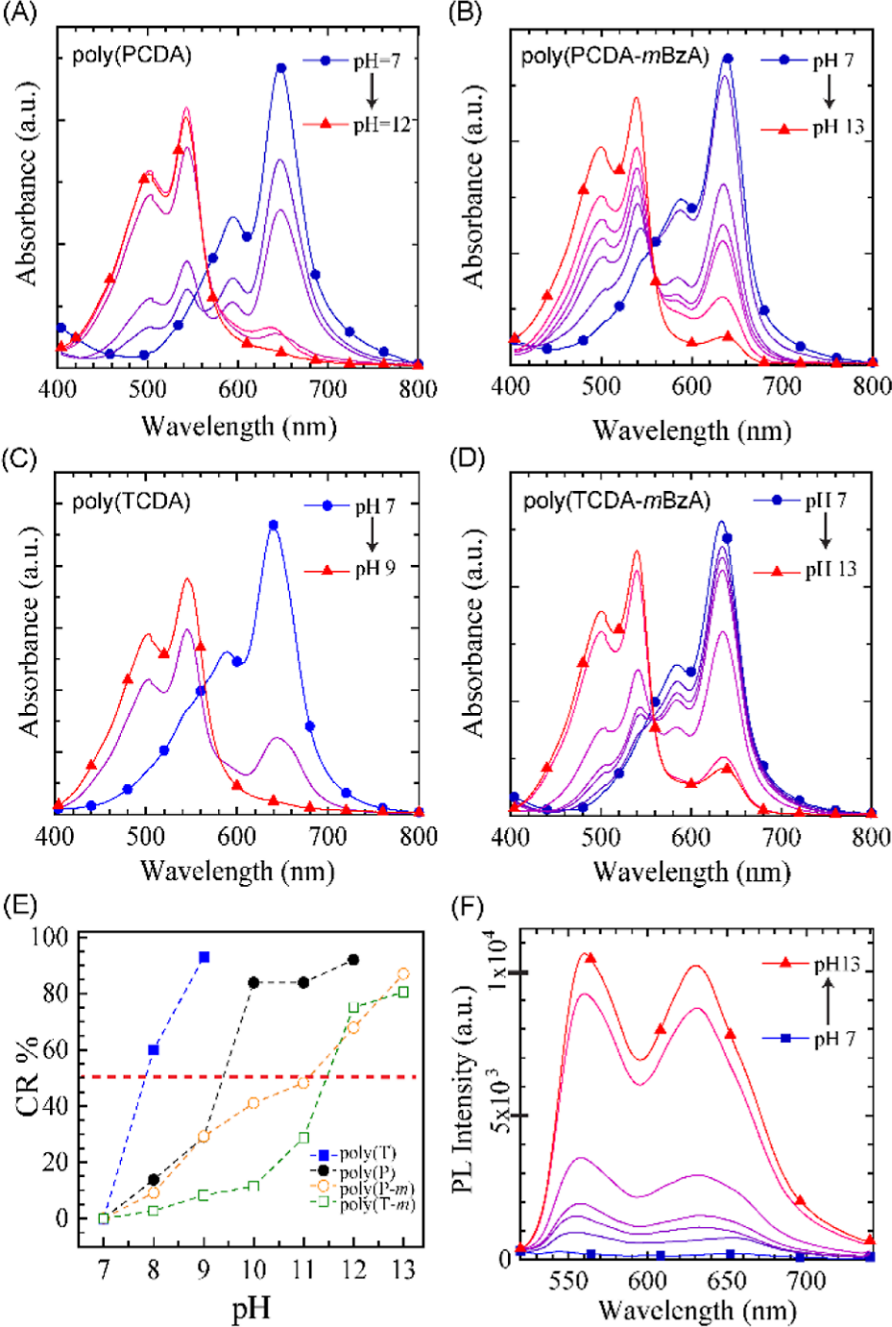
UV–vis absorption spectra of (A) poly(PCDA), (B) poly(PCDA-*m*BzA), (C) poly(TCDA), and (D) poly(TCDA-*m*BzA) photopolymerized in 3 min while pH is increased. (E) CR % of all PDAs with symbols: (box solid, blue) for poly(TCDA), (circle solid, black) for poly(PCDA), (circle open, orange) for poly(PCDA-*m*BzA), and (box, green) for poly(TCDA-*m*BzA). (F) Fluorescence spectra of poly(TCDA-*m*BzA) at an increasing pH.

We recorded their UV–vis absorption spectra to accurately determine the transition pH for individual PDA systems prepared at different photopolymerization durations. We first checked the systems photopolymerized for 3 min (Figure 5A–D). The initial spectra of all solutions at pH 7 show the typical blue-phase PDA absorption peaks with a main peak at ∼640 nm and a shoulder at ∼590 nm. While pH increases, we see the evolution of the red-phase-attributed absorption peaks at around 540 and 500 nm. The spectral transition where the blue-phase bands lose intensity and the red-phase bands start to dominate the spectrum does happen gradually in all polymer solutions but not at the same onset of pH transition. We calculated the colorimetric response percentage (CR %), which can be interpreted as response efficiency, from UV–vis spectra for all PDA solutions and determined the onset of the pH transition at 50% CR (Figure 5E). PDAs obtained from unmodified Das exhibited lower color transition pH values than those of the *m*BzA-substituted counterparts. The poly(TCDA) solution shows color transition pH at the lowest pH value, pH < 7.8, followed by poly(PCDA) with a color transition pH of 9.2. Even though poly(PCDA) and poly(TCDA) possess the same headgroup functionality, they differ in the hydrophobic alkyl chain lengths, where PCDA has 12 and TCDA has 10 carbons.

Therefore, poly(TCDA) assemblies have weaker dispersion forces, which make the entire system easy to transition to the red phase once exposed to an external trigger.^35^ The colorimetric response of the *m*BzA-substituted PDAs to pH increase came at remarkably higher pH, 10.8 for poly(PCDA-*m*BzA) and 11.4 for poly(TCDA-*m*BzA) having CR % values of 87 and 81%, respectively. The delayed pH response of the *m*BzA-substituted PDAs is due to the enhanced intermolecular interactions among the BzA headgroups.^44^ There are additional hydrogen bond interactions due to amide groups and π–π stack interactions among the aromatic groups. When the intermolecular interactions get stronger, the conformation transition of the PDA backbone requires more stress exertion on the backbone.^35,61^ In this system, stress comes at higher basic conditions, meaning higher concentrations of OH^–^ ions; therefore, we see significantly high color transition pH values for modified PDAs.

The blue-phase PDAs do not exhibit fluorescence due to *A*_g_ symmetry, where the singlet excited state demonstrates a dipole-forbidden transition.^62^ However, once the PDA conformation transitions to a twisted, nonplanar red phase, a segmental rearrangement of the polymer molecules occurs with the symmetry that supports radiative decay with the lowest excited state, the *B*_u_ state. Therefore, the PDA system starts to give a fluorescence response to this conformational change in its structure. In Figure 5F, we showed the fluorescence response of poly(TCDA-*m*BzA) to pH change from 7 to 13, whereas the fluorescence spectra of the other PDA systems are given in Supporting Information Figure S8. The fluorescence emission spectra of CPs are more sensitive to any structural change or binding event than the absorption spectra. Figure 5F shows almost zero fluorescence at pH 7. The fluorescence intensity gradually grows at 560 and 630 nm until pH 11. Above pH 11, we see a jump in PL intensity almost 10-fold, reaching 10^4^ at pH 12–13. These results support the color transition pH obtained from the CR % calculation for poly(TCDA-*m*BzA) and other PDAs. Even though DLS might not give reliable size information for our mix morphology containing the PDA system, we checked the change in the size distribution of assemblies to evaluate potential aggregation at highly alkaline conditions. Results show a gradual growth in the size of all PDA assemblies reaching up to micrometer size at high pH conditions with increased polydispersity, indicative of aggregation (Figure S9).

**Figure 6:**
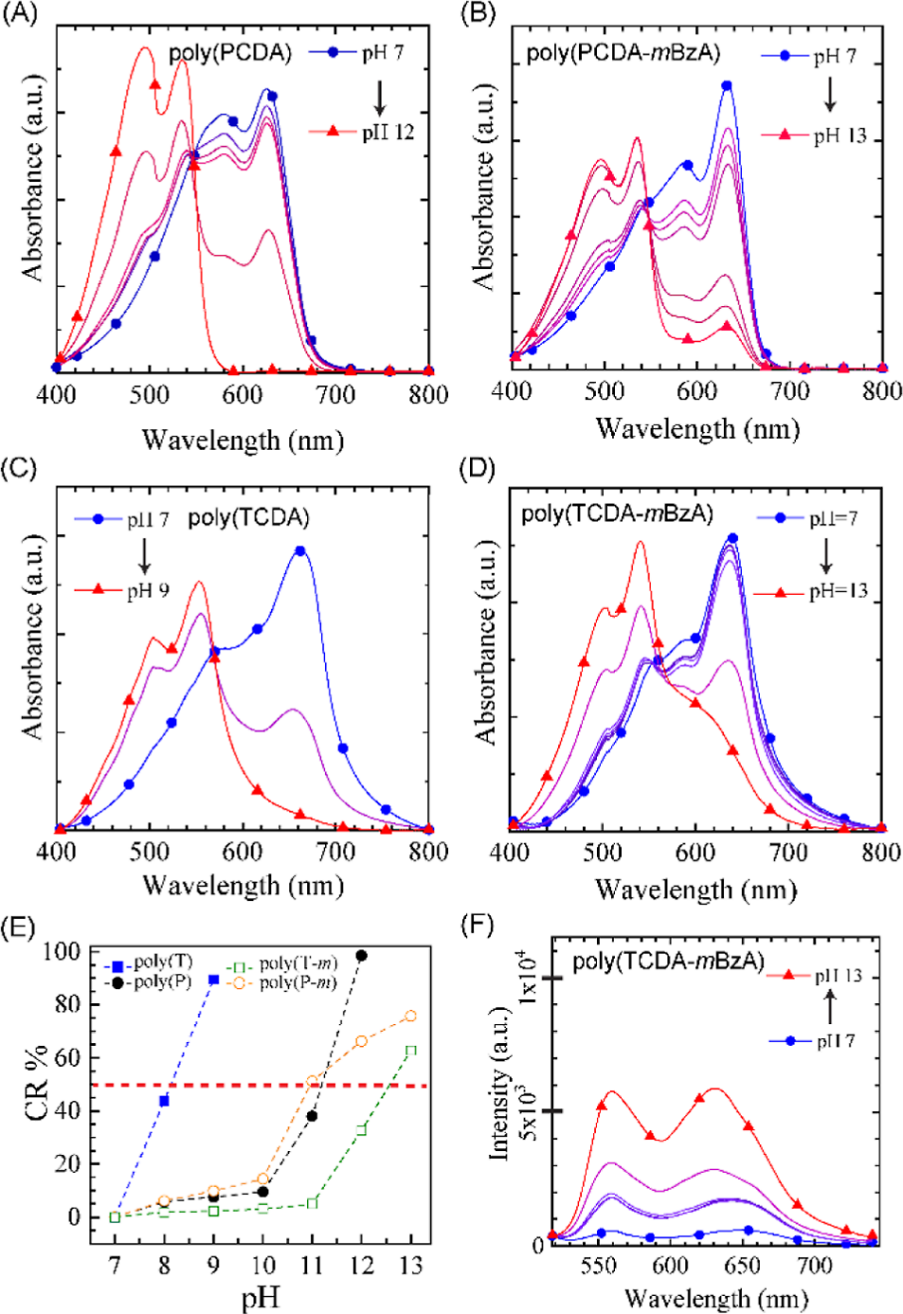
UV–vis absorption spectra of (A) poly(PCDA), (B) poly(PCDA-*m*BzA), (C) poly(TCDA), and (D) poly(TCDA-*m*BzA) photopolymerized in 20 min while pH is increased. (E) CR % of all PDAs with symbols (box solid, blue) for poly(TCDA), (circle solid, black) for poly(PCDA), (circle open, orange) for poly(PCDA-*m*BzA), and (box, green) for poly(TCDA-*m*BzA). (F) Fluorescence spectra of poly(TCDA-*m*BzA) at increasing pH.

The UV–vis absorption spectra of PDAs obtained after 20 min of photopolymerization showed similar transitioning patterns. The absorbance band at 640 nm decreases in intensity, while the band at 540 nm emerges and dominates the spectra at increasing pH (Figure 6A–D). Thus, all PDAs transitioned from the blue to the red phase, similar to the ones photopolymerized for 3 min. However, there is a clear difference in the color transition pH, where the onset has shifted to higher pH values for all systems (Figure 6E). The extended photopolymerization duration increased the required dose of stress exertion on the backbone to trigger a segmental realignment, thus a phase transition.^25,52^ The color transition pH for poly(PCDA) has risen to pH 11 from 9.2, whereas the change was not as dramatic for the poly(TCDA) system, which experienced a slight increase from pH 7.8 to pH 8.2. The modified PDAs show the color transition pH shifted from 10.8 to 11 for poly(PCDA-*m*BzA) and 11.4 to 12.5 for poly(TCDA-*m*BzA). The higher color transition pH of poly(TCDA-*m*BzA) compared to poly(PCDA-*m*BzA) might be due to the difficulty in inducing stress relief to the backbone in shorter alkyl chain segments when a bulky headgroup is present. This could originate from local interaction changes and backbone movement limitations.^63^ Although modified PDAs showed color transition, we recorded their CR % reaching only up to 65 and 70% for poly(TCDA-*m*BzA) and poly(PCDA-*m*BzA), respectively, in Figure 6E. Such CR % levels indicate a decreased colorimetric response efficiency in modified PDAs produced after long photopolymerization. This decrease was not observed for the unmodified PDAs produced after longer photopolymerization times.

We performed fluorescence emission measurements of PDAs photopolymerized for 20 min to cross-check again the CR % obtained from UV–vis spectra. Figure 6F shows the poly(TCDA-mBzA) spectra during the pH change from 7 to 13, and the spectra of other PDA systems are shown in Supporting Information Figure S10. The system produced after longer photopolymerization exhibits a different fluorescence intensity growth from the shortly photopolymerized ones. The PL intensity at bands around 560 and 630 nm emerges at pH 8 and stays at that intensity level until pH 12. We recorded a noticeable growth in PL intensity at pH 12 and especially at pH 13. However, the final PL intensity reached in this system is only half that the shortly photopolymerized system reached. This outcome supports the 65% response efficiency calculated from the absorption spectrum of the pH 13 system. A similar result is also obtained for poly(PCDA-*m*BzA), which reached 70% response efficiency at pH 13. Clearly, OH^–^ ions added into the system to reach pH 13 were insufficient to exert the stress required for the complete conformation transition of modified PDAs. A transition limit exists when H-bonding interactions are enhanced among terminal groups in the PDA structure.

Finally, we checked the change in the size distribution of PDA assemblies obtained after prolonged UV exposure with DLS. Results obtained for all PDA assemblies except poly(TCDA-*m*BzA) showed very subtle changes in the size distribution at high alkaline conditions (Figure S11). Longer exposure to UV irradiation during polymerization led to systems that retained structural integrity under high pH conditions, whereas shorter UV irradiated ones could not. This might also be due to higher polymer conversion in longer UV-exposed systems.

### Reversible Colorimetric pH Response of PDAs

**Figure 7:**
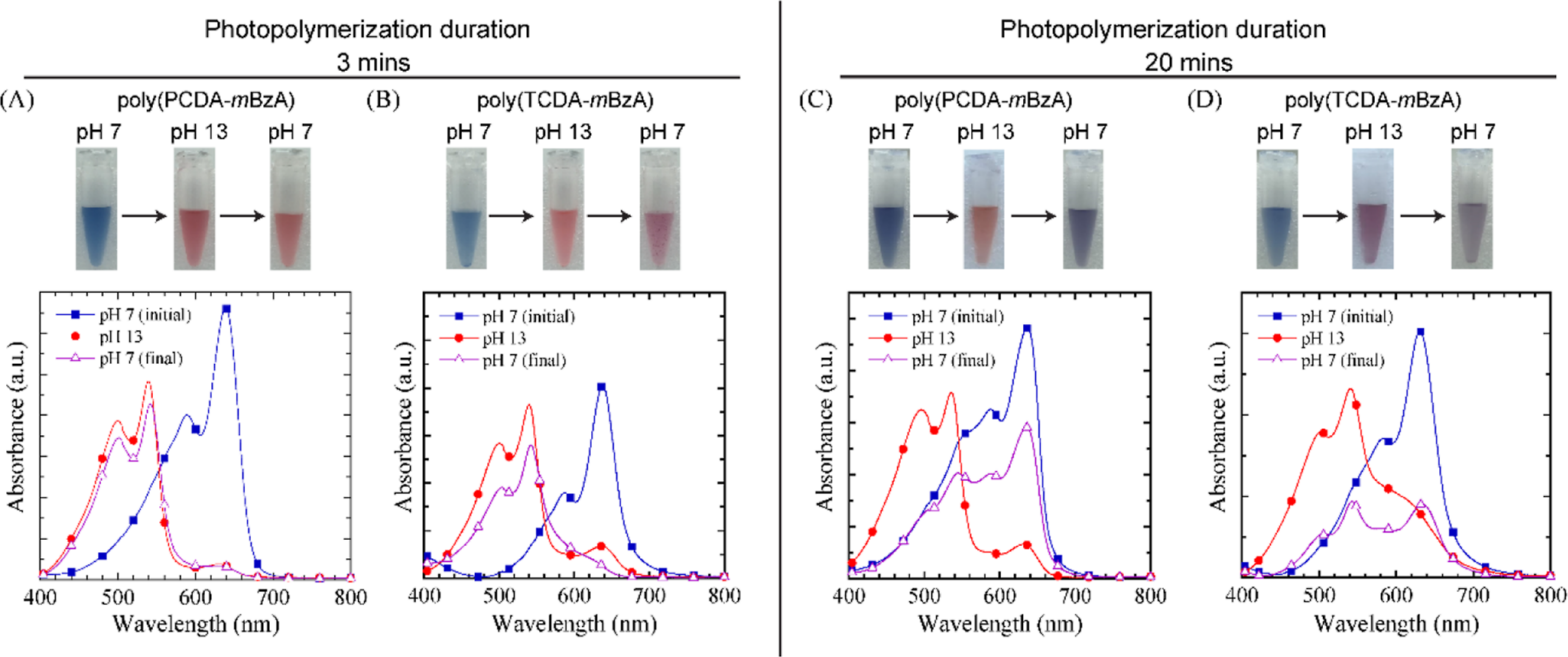
Photos and corresponding absorption spectra of PDA solutions during reversibility experiments, starting at pH 7 (initial), increasing to 13 and decreasing back to pH 7 (final). (A) Poly(PCDA-*m*BzA) and (B) poly(TCDA-*m*BzA) photopolymerized in 3 min. (C) Poly(PCDA-*m*BzA) and (D) poly(TCDA-*m*BzA) photopolymerized in 20 min.

The conformation transition of PDAs possessing monocarboxylic headgroups, such as poly(PCDA) and poly(TCDA), from the planar blue phase to the twisted, nonplanar red phase is irreversible.^44,64^ The amide, aromatic, or carboxylic acid groups in the *m*BzA headgroup create cooperative, stable, and strong H-bonding. We checked whether structural modifications were sufficient to obtain a reversible pH response from the modified PDAs. We designed the reversibility experiments to start with PDA solutions at pH 7, increase the pH to 13 by adding concentrated KOH solution, and drop back to pH 7 by adding concentrated HCl solution. Figure 7A shows the images and the corresponding absorption spectra of poly(PCDA-*m*BzA) photopolymerized in 3 min during the reversibility experiment. Results showed the colorimetric response of PDAs from blue to red when the pH was increased from 7 to 13 but not the reverse response when the pH was dropped to 7. The characteristic red-phase absorption peaks remained with only a slight drop in intensity due to the dilution effect. Figure 7B shows almost the same result obtained for the poly(TCDA-*m*BzA) photopolymerized in 3 min. Thus, neither of the modified systems shows a reversible colorimetric response to pH change.

Structural alterations did not solely enable a conformational transition back to the blue phase. Therefore, we checked whether reversibility could be achieved by increasing the photopolymerization time to 20 min. Poly(PCDA-*m*BzA) undergoes a blue to red transition upon pH increase. Interestingly, they also exhibit reversibility when the pH drops to 7 (Figure 7C). The absorption spectra recorded when pH was brought back to 7 showed the blue-phase characteristic bands with a reduced intensity due to the dilution effect. Similarly, Figure 7D shows poly(TCDA-*m*BzA) undergoing a reversible colorimetric response to pH changes from 7 to 13 and back to 7. However, this reverse response is not from red back to blue but to the purple phase, which exhibits both the blue-phase-attributed absorption band at 630 nm and the red-phase-attributed band at around 530 nm in its spectrum. The intensities of both bands are almost equal but lower than the rest due to the dilution effect. While poly(PCDA-*m*BzA) polymerized under 20 min of UV exposure exhibits full recovery of the blue-phase band, thereby the planar conformation, poly(TCDA-*m*BzA) could not. Instead, the conformation transition of poly(TCDA-*m*BzA) stops at the twisted, ribbon-like intermediate purple-phase conformation. The reason behind the different reversibility behavior of the same headgroup carrying PDA systems might be the presence of stronger dispersion forces in poly(PCDA-*m*BzA) due to the longer alkyl tail promoting the polymer chain reorganization to its initial planar blue phase. Moreover, in the previous section, we showed that poly(TCDA-*m*BzA) obtained after 20 min of UV exposure had the lowest CR % around 65%, followed by poly(PCDA-*m*BzA) at 70%. This indicates a limitation in the system against conformation transition upon exposure to high pH. We believe that modified PDAs possess a mixture of twisted, nonplanar, and minority planar conformations at pH 13. This mixture of conjugated backbone conformations provides enough freedom for chains to reverse the backbone conformation to the thermodynamically unfavorable planar blue one. Despite having weaker dispersion interactions, the poly(TCDA-*m*BzA) system experiences either a resistance against a complete conformation change or a dissipation pathway for the strain induced on the chains, preventing it from obtaining a fully planarized conformation. A similar result, the intermediate purple phase, was obtained earlier in another TCDA derivative PDA system against different triggers.^35^ Modified TCDA-based PDA systems should be further studied to fully understand the mechanisms behind the intermediate purple-phase formations fully.

## Conclusions

In this study, we aimed to obtain the reversible colorimetric response to a pH change. To achieve that, we tailored DAs with enhanced H-bonding interactions among headgroups and tuned the photopolymerization time. The synergy of both mechanisms enabled the reversible response to pH under basic conditions. The structural modification on the monomer with a bulky headgroup reduced the freedom of chains once they were self-assembled and photopolymerized. The limitation of conformational realignments in these systems revealed reduced photopolymerization efficiency and pH sensitivity. A reduced colorimetric response efficiency to pH change was observed once the samples were exposed to long photopolymerization times. Results indicate a system consisting of a conformation blend, where the nonplanar red phase coexists with the planar blue phase (minor) in a high pH environment. The conformational blend enables the system to overcome the thermodynamic restrictions of transitioning from a stable to a metastable conformation. Thereby, it becomes instrumental in obtaining a reversible colorimetric pH response. Exposure to UV for extended periods and enhanced H-bond interactions provide the restorative force to transition back to either the planar blue phase or the intermediate purple phase upon recovering the pH to the initial condition. Our study brings an in-depth understanding of supramolecular interactions acting in self-assembling PDA systems and offers a new perspective on obtaining reversibility by combining different factors.

## Supporting Information Available

^1^H NMR, ^13^C NMR, and FTIR spectra of modified diacetylene monomers; size distribution of assemblies photopolymerized at different UV exposure times and pH conditions; and fluorescence spectra of polymer solutions at different pH conditions (PDF)

## Abbreviations

CPs: conjugated polymers
PDAs: polydiacetylenes
DA: diacetylene
BzA: benzoic acid
PCDA: 10,12-pentacosadiynoic acid
TCDA: 10,12-tricosadiynoic acid
FTIR: Fourier transform infrared
ATR: attenuated total reflectance
DLS: dynamic light scattering
