# Supplementary material for: Effect of UV Irradiation Time and Headgroup Interactions on the Reversible Colorimetric pH Response of Polydiacetylene Assemblies
Source: ACS Omega. 2023 Sep 26;8(40):37213–24. doi: 10.1021/acsomega.3c04845 (PMC10568583; doi:10.1021/acsomega.3c04845)
Supplement: Supplementary file 1 — ao3c04845_si_001.pdf [file ao3c04845_si_001.pdf]

SUPPLEMENTARY DOCUMENT

The effect of UV-irradiation time and headgroup interactions on the reversible colorimetric pH response of polydiacetylene assemblies

Gizem Beliktay[b], Tayyaba Shaikh[a], Emirhan Koca [a], Hande E. Cingil [a,]

[a]Sabanci University Nanotechnology Research and Application Center, Istanbul 34956, Turkey

[b]Faculty of Engineering and Natural Sciences, Sabanci University, Istanbul 34956, Turkey

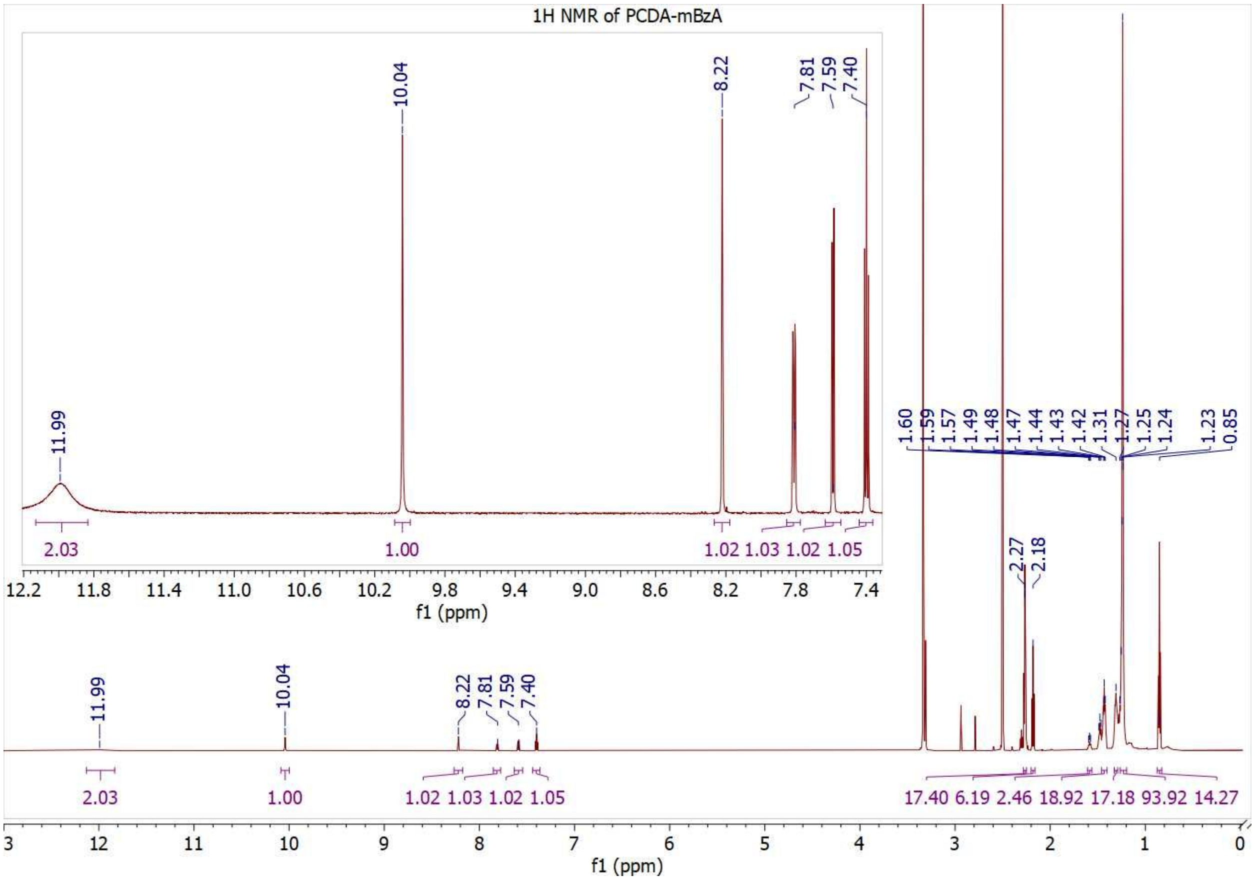

Figure. S1. ¹H NMR spectrum of **PCDA-*m*BzA**

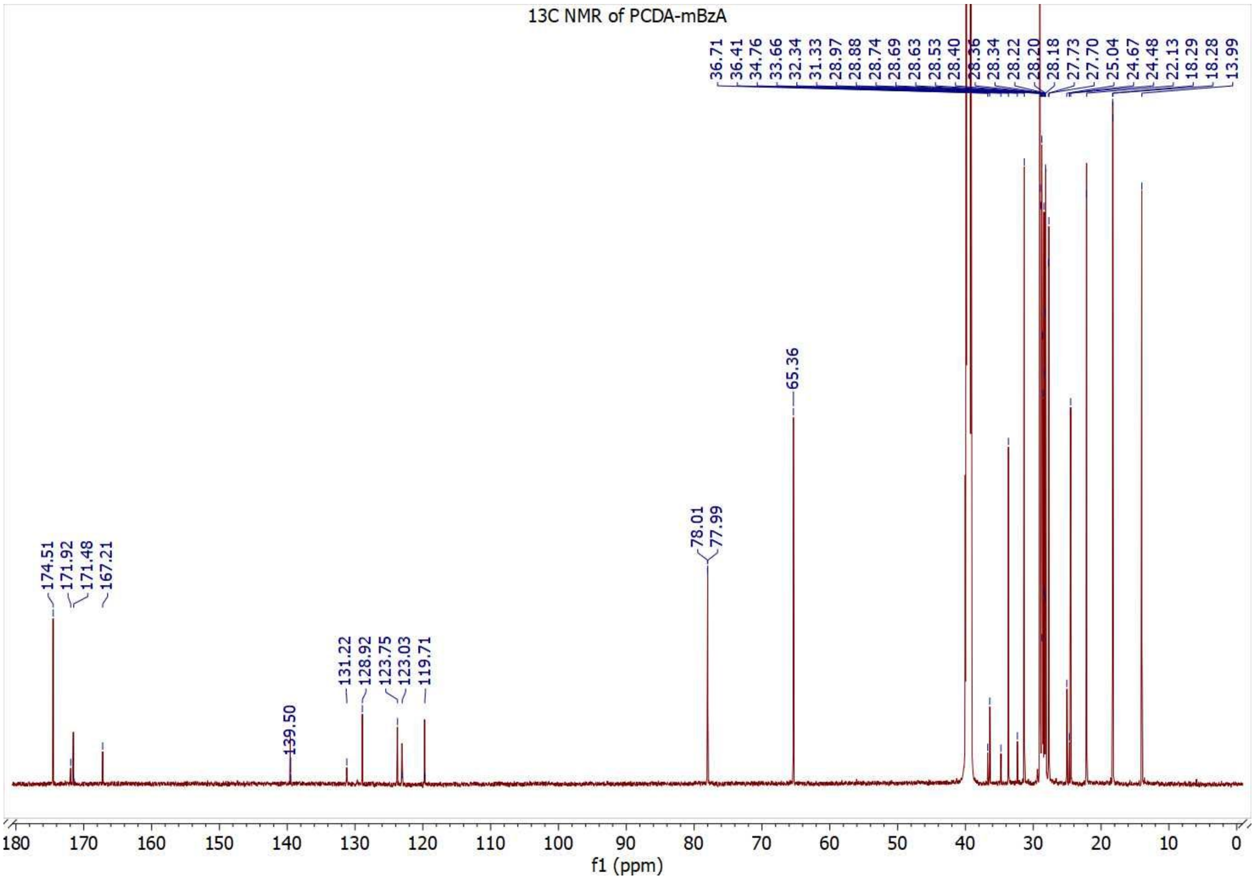

Figure. S2. ^{13}C NMR spectrum of **PCDA-*m*BzA**

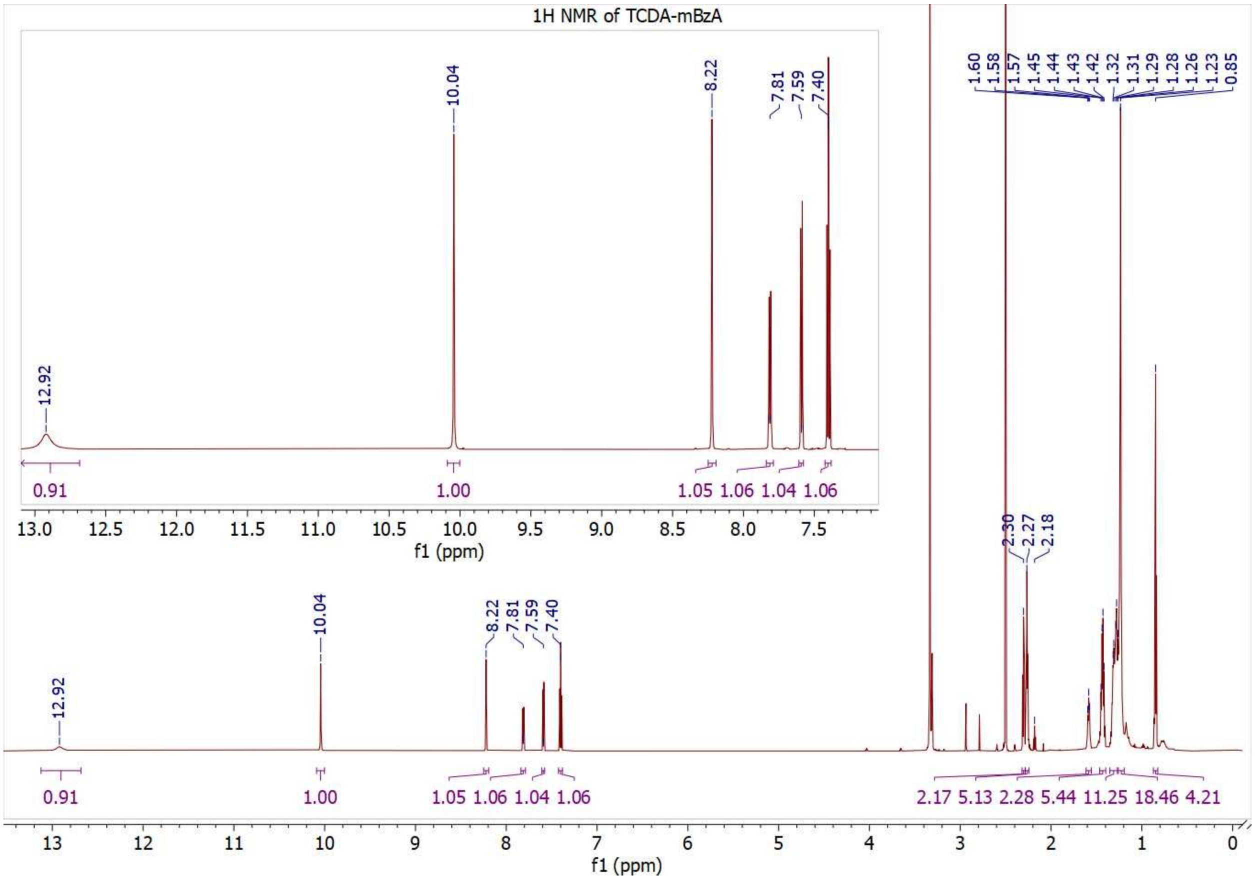

Figure. S3. ^{1}H NMR spectrum of **TCDA-*m*BzA**

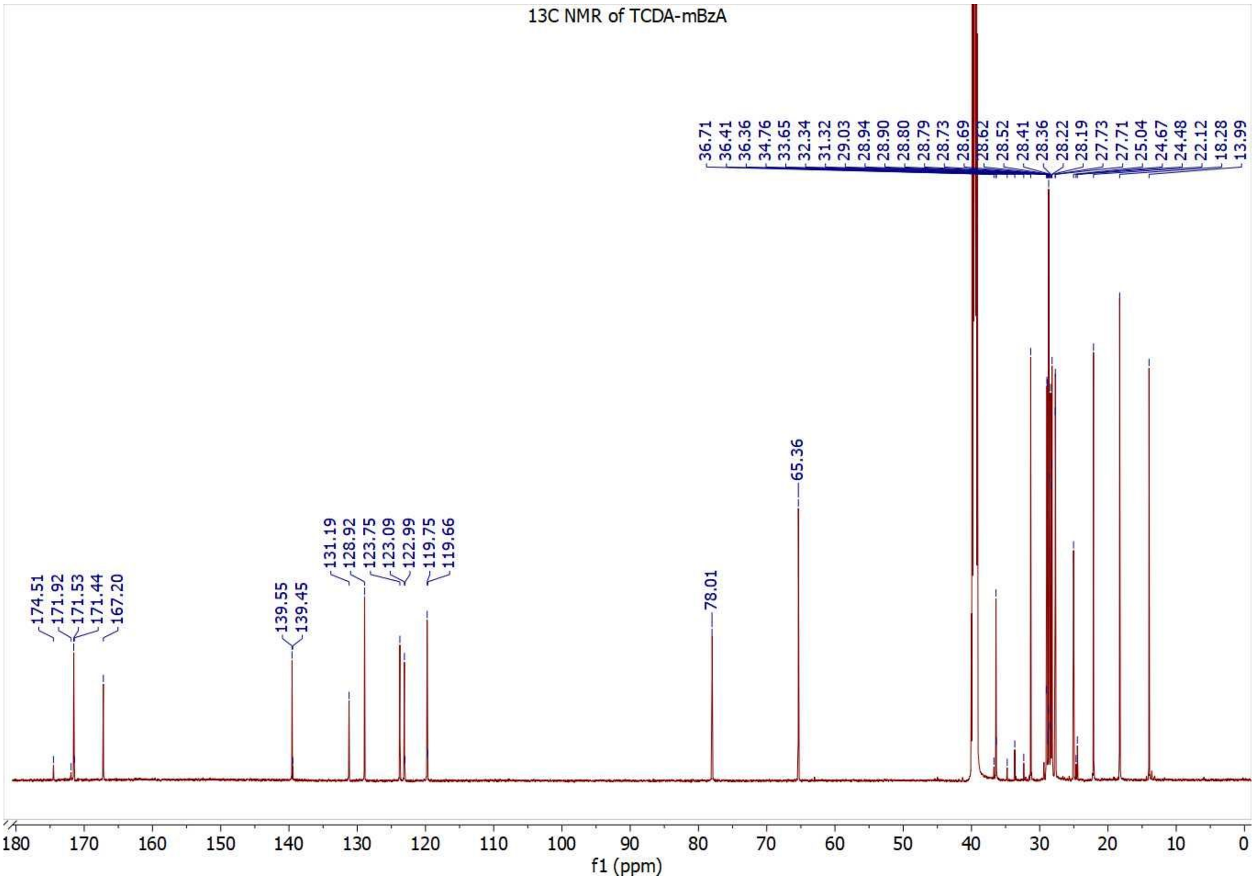

Figure. S4. ^{13}C NMR spectrum of **TCDA-*m*BzA**

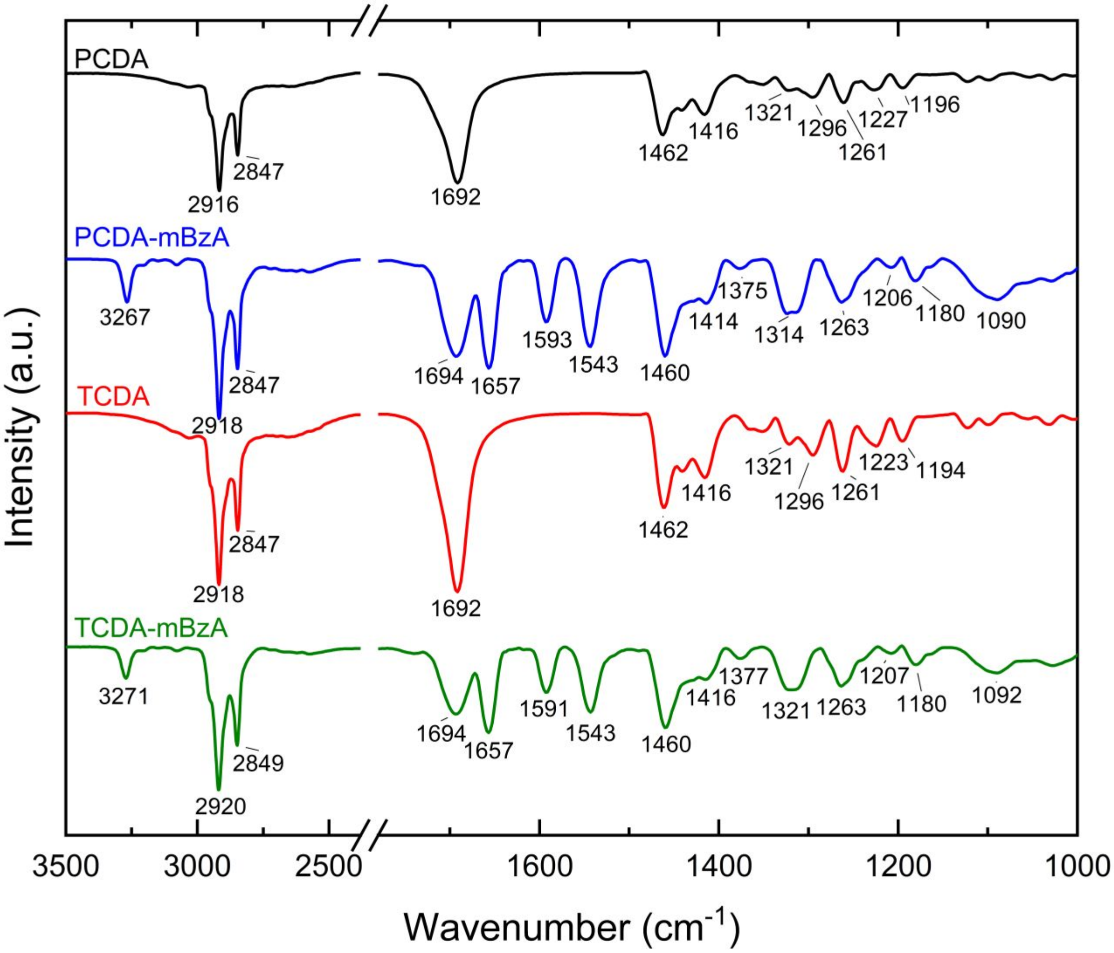

Figure. S5. FTIR spectra of the DA monomers.

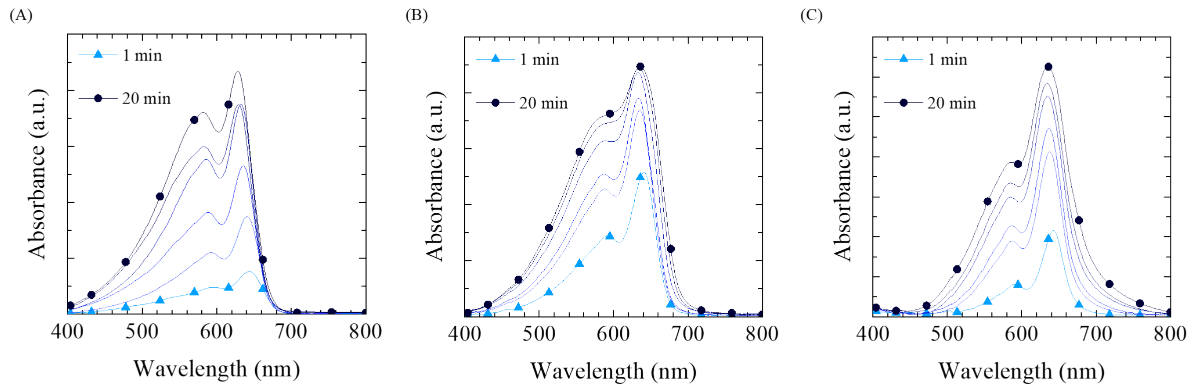

Figure. S6. UV-Vis absorption spectra of PDA solutions obtained after varying photopolymerization durations from 1 to 20 minutes for (A) poly(PCDA), (B) poly(TCDA) and (C) poly(TCDA-*m*BzA).

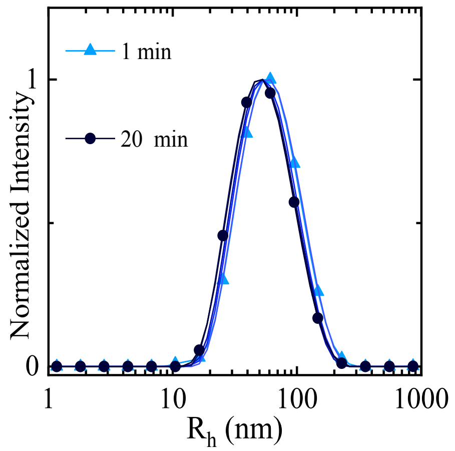

Figure. S7. The normalized intensity weighted size distributions displaying the mean hydrodynamic radius (R_h) of poly(PCDA-*m*BzA) obtained at different photopolymerization durations.

Table S1. Hydrodynamic Radii (R_h) values and polydispersity index (PDI) of PDAs photopolymerized after 1 to 20 minutes

	HYDRODYNAMIC RADII (R_H) (NM)			
Uv Irradiation Time (Mins)	poly(PCDA)	poly(PCDA-*m*BzA)	poly(TCDA)	poly(TCDA-*m*BzA)
**1**	46.4	67.8	73.9	151.9
**3**	48.0	68.3	68.2	132.8
**5**	49.1	64.3	66.8	131.2

**10**	50.7	61.4	64.4	132.6
**15**	49.1	60.6	64.4	124.8
**20**	45.9	60.3	63.4	125.2

PDI

Uv Irradiation Time (Mins)	poly(PCDA)	poly(PCDA-*m*BzA)	poly(TCDA)	poly(TCDA-*m*BzA)
**1**	0.29	0.27	0.21	0.25
**3**	0.30	0.30	0.23	0.24
**5**	0.33	0.36	0.22	0.24
**10**	0.52	0.33	0.22	0.26
**15**	0.47	0.31	0.22	0.25
**20**	0.50	0.38	0.23	0.25

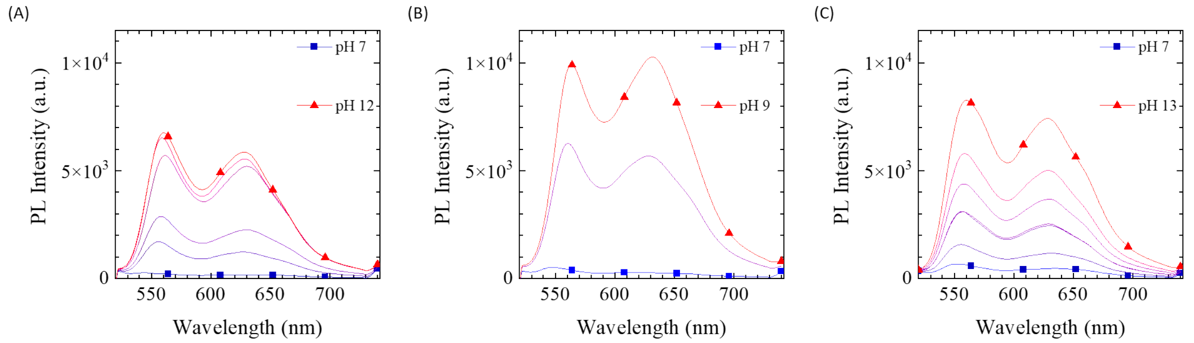

Figure. S8. Fluorescence spectra of PDA solutions photopolymerized for 3 minutes at varying pH from 7 to 13 for (A) poly(PCDA), (B) poly(TCDA) and (C) poly(PCDA-*m*BzA).

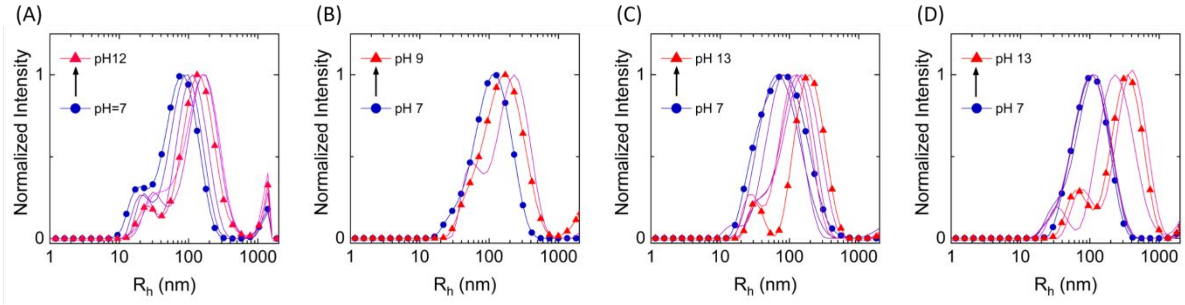

Figure. S9. The normalized intensity weighted size distributions displaying the mean hydrodynamic radius (R_h) of PDA solutions photopolymerized for 3 minutes at varying pH from 7 to 13 for (A) poly(PCDA), (B) poly(TCDA), (C) poly(PCDA-*m*BzA) and (D) poly(TCDA-*m*BzA).

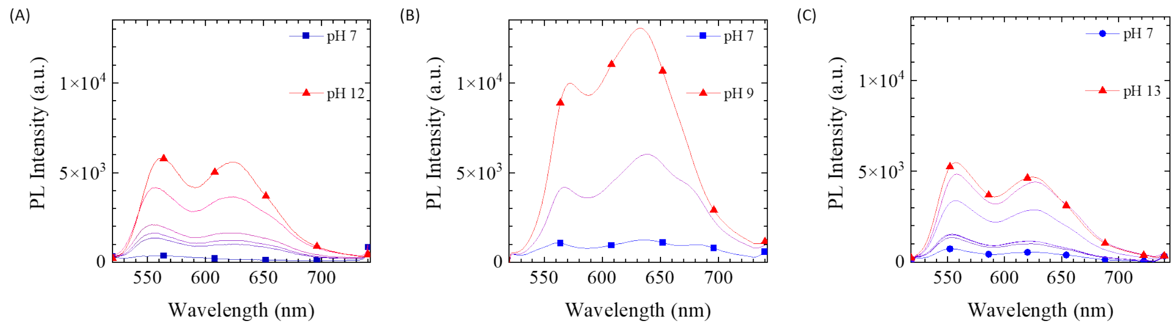

Figure. S10. Fluorescence spectra of PDA solutions photopolymerized for 20 minutes at varying pH from 7 to 13 for (A) poly(PCDA), (B) poly(TCDA) and (C) poly(PCDA-*m*BzA).

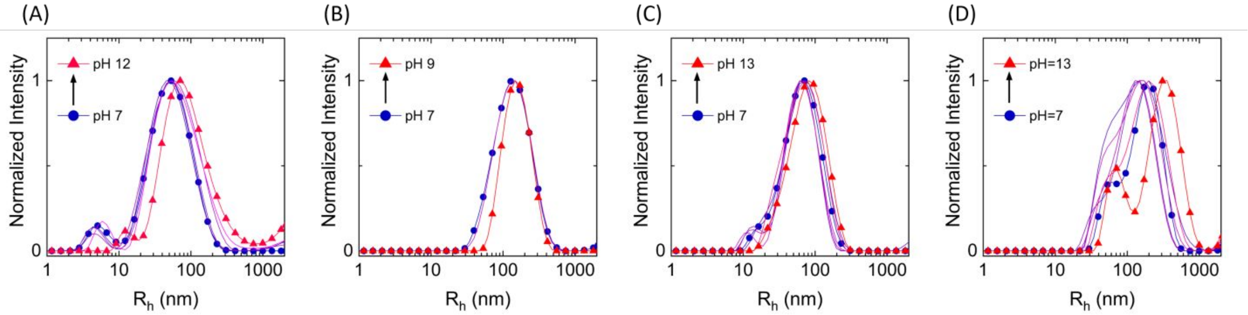

Figure. S11. The normalized intensity weighted size distributions displaying the mean hydrodynamic radius (R_h) of PDA solutions photopolymerized for 20 minutes at varying pH from 7 to 13 for (A) poly(PCDA), (B) poly(TCDA), (C) poly(PCDA-*m*BzA) and (D) poly(TCDA-*m*BzA).
